# ColorDetect RT-LAMP Assay for the Rapid, Sensitive, and Specific Detection of Porcine Abortion-Associated Pestivirus (PAAPeV)

**DOI:** 10.3390/vetsci13010074

**Published:** 2026-01-12

**Authors:** Xu Yang, Ying Li, Wenqi Yin, Wenjie Tang, Hui Diao, Mengjia Zhou, Hao Yang, Wenyi Fu, Lu Yong, Xu Luo, Guo Liao, Yuancheng Zhou

**Affiliations:** 1 Animal Genetic Breeding and Reproduction Key Laboratory of Sichuan Province, Sichuan Animal Science Academy, Chengdu 610066, China; 2 Sichuan Provincial Engineering and Technology Research Center for Animal Biologics, Animtech Bioengineering Co., Ltd., Chengdu 610299, China; 3 Agriculture and Rural Affairs Bureau of Jingyang District, Deyang 618000, China

**Keywords:** Porcine abortion-associated pestivirus (PAAPeV), reverse transcription loop-mediated isothermal amplification (RT-LAMP), RT-qPCR, rapid detection

## Abstract

The primary objective of this study was to develop and optimize a colorimetric reverse transcription loop-mediated isothermal amplification (RT-LAMP) assay for porcine abortion-associated pestivirus (PAAPeV)—an emerging pathogen that causes severe reproductive disorders in swine, for which no effective treatments or vaccines are currently available. In this study, four sets of LAMP primers were designed and screened for the colorimetric RT-LAMP assay, targeting the highly conserved 5′ untranslated region (5′UTR) of PAAPeV. Three reaction parameters, including reaction temperature, reaction duration, and inner-to-outer primer ratio, were then optimized based on cycle threshold (Ct) values, fluorescence intensity, and color changes of the endpoint products. Subsequently, the specificity and sensitivity of the optimized colordetect RT-LAMP assay were systematically validated, and its diagnostic performance was compared with that of the gold-standard reverse transcription quantitative polymerase chain reaction (RT-qPCR). The results demonstrated that the optimized assay achieved a detection limit of 2 copies/μL under the conditions of 65 °C incubation for 25 min and an inner-to-outer primer ratio of 8:1, with results amenable to naked-eye interpretation. Furthermore, this assay exhibited high specificity, showing no cross-reactivity with other known pestiviruses or prevalent swine pathogens. Clinical sample testing results showed 100% concordance between colordetect RT-LAMP and RT-qPCR. Collectively, this colordetect RT-LAMP assay represents a rapid, sensitive, and specific tool for PAAPeV RNA detection in both clinical laboratories and field settings.

## Simple Summary

Emerging porcine pestivirus-associated diseases continue to pose significant challenges to preventive and control measures within the global swine industry. In this study, we developed a reverse transcription loop-mediated isothermal amplification (RT-LAMP) assay for a newly identified pestivirus—porcine abortion-associated pestivirus (PAAPeV)—designated as colordetect RT-LAMP. Based on the highly conserved 5′ un-translated region (5′UTR) of PAAPeV, this assay achieved a detection limit of 2 copies/μL under 65 °C incubation for 25 min, and had no cross-reactivity against other known pestiviruses or prevalent swine pathogens. Clinical validation revealed 100% concordance between the colordetect RT-LAMP assay and the gold-standard RT-qPCR method. This assay serves as a rapid, sensitive, and specific diagnostic tool for resource-limited settings and thus holds substantial promise for widespread deployment in on-site and regional veterinary laboratories.

## 1. Introduction

Porcine abortion-associated pestivirus (PAAPeV) is a newly identified member of the Pestivirus genus in the Flaviviridae family, isolated from clinical samples of aborted fetuses and piglets exhibiting congenital tremors in China in 2023 [1]. Similar to known Pestiviruses, PAAPeV consists of positive-sense, single-stranded RNA with a roughly 12.5 kb genome length containing a single open reading frame (ORF) that encodes a polyprotein flanked by 5′ and 3′ untranslated regions (UTRs) [1]. The 5′UTR is highly conserved across pestiviruses and functions as an internal ribosomal entry site for translation initiation of the pre-polyprotein and genome replication [2,3].

Pestiviruses can infect a diverse range of hosts, including swine, ruminants, bats, and rodents, resulting in considerable economic losses in husbandry [3,4,5]. As is widely recognized, bovine viral diarrhea virus (BVDV) infection in cattle typically results in clinical manifestations including fever, mild diarrhea, and abortion, while classical swine fever virus (CSFV) infection in swine induces fever, neurological disorders, hemorrhagic symptoms, and high mortality rates. Collectively, both of them impose substantial pressure on livestock industry worldwide [6,7]. As a newly identified member of the genus *Pestivirus*, it has been reported that PAAPeV could infect both porcine and murine species, highlighting its potential for cross-species transmission across diverse mammalian hosts [1]. Therefore, the potential impact of PAAPeV infection on the swine industry warrants serious attention, and it is necessary to develop a rapid, efficient and convenient detection method for PAAPeV.

Rapid and accurate diagnosis is essential to the successful control of any animal disease [8]. For members of the genus Pestivirus such as CSFV and BVDV, current diagnostic methods include virus isolation, fluorescent antibody technique, enzyme-linked immunosorbent assay (ELISA), reverse transcription polymerase chain reaction (RT-PCR), and quantitative RT-PCR (RT-qPCR) [7,9,10,11,12,13]. Although RT-PCR and RT-qPCR are widely recognized for their high sensitivity and specificity in viral RNA detection, the requirements of complex operation and costly specialized equipment limit their practicality for on-site diagnostic applications. In recent years, numerous studies have validated the potential utility of loop-mediated isothermal amplification (LAMP) and reverse transcription LAMP (RT-LAMP) assays for the rapid detection of viral nucleic acids (DNA or RNA) [14,15,16,17,18,19,20,21]. Given that the 5′UTR is highly conserved among pestiviruses, it has been recognized as a preferred target region for the detection of BVDV and CSFV RNA [6,20]. However, as a newly identified member of the genus *Pestivirus*, no RT-LAMP assays for PAAPeV detection have been reported to date.

The primary objective of this study was to develop and optimize a colorimetric RT-LAMP assay for the early identification and continuous monitoring of PAAPeV, thereby facilitating timely intervention to prevent the outbreak and transmission of its infections. Herein, we developed a specific and reliable RT-LAMP assay for the rapid detection of PAAPeV. Specifically, a set of six primers was designed to amplify the target region within the 5′UTR of the PAAPeV genome, and this approach yielded highly sensitive and specific results without the need for expensive instruments or reagents.

## 2. Materials and Methods

### 2.1. Viruses Strains and Clinical Samples

Classical Swine Fever Virus (CSFV strain C, GenBank accession No: AF531433.1), Bovine Viral Diarrhea Virus type 1 (BVDV1 strain Oregon CV24, Strain accession No: CVCC AV69, China), Porcine Reproductive and Respiratory Syndrome Virus type 1 (PRRSV1 strain SC-2020-1, GenBank accession No: MW115431.1), Porcine Reproductive and Respiratory Syndrome Virus type 2 (PRRSV2 strain SC/DJY, GenBank No: MT075480.1; strain SC2012, GenBank accession No: KM189443.1), Pseudorabies virus (PRV strain XJ, GenBank accession No: MW893682.1), Porcine Epidemic Diarrhea Virus (PEDV strains CV777, GenBank accession No: AF353511.1), Porcine Circovirus type 2 (PCV2 Strain SCABTC-PCV2-YQL, GenBank accession No: PQ123995.1), Porcine Circovirus type 3 (PCV3 CN, GenBank accession No: PV700519.1), Mammalian Orthoreovirus (MRV strain SC-A, GenBank accession No: DQ396804.1~DQ396806.1), Transmissible Gastroenteritis Virus (TGEV strain SC2021, GenBank accession No: ON858825.1), Porcine Parvovirus (PPV strain NADL-2, GenBank accession No: NC_001718.1) and Japanese Encephalitis Virus (JEV strain SC/2016-3, GenBank accession No: KX779522.1) were preserved in Animal Genetic Breeding and Reproduction Key Laboratory of Sichuan Province. Porcine Rotavirus A (PRoVA strain JY, G9P[23]) was isolated and kept in our laboratory as well. Atypical porcine pestivirus-positive samples and PAAPeV were kindly provided by Professor Zhiwen Xu from Sichuan Agricultural University, Chengdu, Sichuan Province, China.

A total of 54 clinical samples included 24 clinical PAAPeV-positive samples which were kindly provided by Professor Zhiwen Xu (Sichuan Agricultural University). In addition, 30 clinical samples (including brains, lungs, spleen, and liver) were collected from aborted fetuses and piglets presenting with congenital tremors from pig farm in Sichuan province. To prepare the templates, clinical tissue samples were homogenized into a homogeneous slurry with phosphate-buffered saline (PBS, pH 7.4) at a ratio of 1:3 (*w*/*v*). Following three cycles of freeze-thawing (freezing at −80 °C for 30 min and thawing at room temperature), the mixtures were centrifuged at 12,000× *g* for 10 min at 4 °C. The supernatant was collected, and total viral RNA was extracted using FastPure Viral DNA/RNA Mini Kit (Vazyme Biotech Co., Ltd., Nanjing, China) following the manufacturer’s instructions, which was stored at −20 °C for further use.

### 2.2. Sequence Alignment and Phylogenetic Analysis

A multiple sequence alignment of 5′UTR was performed for 25 pestiviruses and PAAPeV from the NCBI database. Phylogenetic trees were constructed using the Neighbor-Joining method for 1000 bootstrap replications in MEGA software (Mega Limited, Auckland, New Zealand). MEGA software and MegAlign software (DNASTAR Inc., Madison, WI, USA) were employed for the 5′UTR nucleotide sequences of PAAPeV, BVDV, CSFV, and APPV sequence alignment.

### 2.3. ColorDetect RT-LAMP Primer Design and Screen

Four sets of LAMP primers were designed based on the 5′UTR of PAAPeV (GenBank Accession No.: PP663643.1) using the NEB Primer Design Tool (https://lamp.neb.com/, accessed on 10 February 2025)), and all primers were synthesized by Sangon Biotech Co., Ltd. (Shanghai, China) (Table 1). Each primer set consisted of six primers, including an outer forward primer (F3), outer backward primer (B3), forward inner primer (FIP), backward inner primer (BIP), forward loop primer (LoopF), and backward loop primer (LoopB). The optimal primer set was selected for subsequent experiments based on three key criteria: cycle threshold (Ct) value, fluorescence intensity, and colorimetric change of the end product.

### 2.4. ColorDetect RT-LAMP Reaction Condition Optimization

The ColorDetect RT-LAMP assay was performed using the ColorDetect LAMP/RT-LAMP 2× Master Mix (Vazyme Biotech Co., Ltd., Nanjing, China). The standard reaction system (25 μL) comprised 12.5 μL of 2× Master Mix, a primer mixture (1.6 μM FIP, 1.6 μM BIP, 0.2 μM F3, 0.2 μM B3, 0.4 μM LoopF, and 0.4 μM LoopB), 1 μL of template, and RNase-free double-distilled water (ddH_2_O) to make up the final volume. The reaction mixture was initially incubated at 65 °C for 25 min as a preliminary condition.

To determine the optimal assay parameters, three critical reaction conditions were systematically optimized: (1) reaction temperature (60 °C, 61 °C, 62 °C, 63 °C, 64 °C, and 65 °C); (2) reaction duration (5, 10, 15, 20, 25 and 30 min); and (3) inner-to-outer primer ratio (1:1, 2:1, 4:1, and 8:1). After amplification using CFX Connect Real-Time System (Bio-RAD, Hercules, CA, USA), the assay results were evaluated based on three metrics: Ct values, fluorescence intensity, and colorimetric changes of the endpoint products.

### 2.5. Specificity and Sensitivity Assays

To evaluate the specificity of the colordetect RT-LAMP assay, reactions were performed using RNase-free ddH_2_O as the negative control, with nucleic acids from TGEV, BVDV, PEDV, PCV2, CSFV, PRRSV, MRV, JEV, PPV, PoRV, PCV3, APPV and PRV serving as templates. Specificity was determined based on the colorimetric changes of the endpoint products.

To assess the sensitivity of the colorimetric RT-LAMP assay, pUC57 plasmids bearing the PAAPeV 5′UTR sequence was constructed and synthesized (Sangon Biotech Co., Ltd., Shanghai, China). Then, a tenfold dilution series of pUC57-PAAPeV 5′UTR plasmid ranging from 2 × 10^5^ to 2 × 10^−1^ copies/µL were tested in duplicate using optimized colordetect RT-LAMP assay and qPCR using SYBR Green qPCR kit (Beyotime Co., Ltd., Shanghai, China) for comparison.

### 2.6. RT-qPCR

The TaqMan probe-based RT-qPCR assay for the detection of PAAPeV was performed as described previously [1]. Briefly, the reaction system (25 μL total volume) comprised 12.5 μL of Premix Ex Taq (Probe RT-qPCR) (Takara, Dalian, China), 1 μL (10 μM) of each primer (Forward: 5′-CAC GGA GCT ACC AAG GAA AT-3′; Reverse: 5′-GGA AAT GCT AGG GTG CTT AAC-3′), 0.2 μL (10 μM) of probe (BHQ1-TGG AAG CAA TAG GGA GGC ACA AGA-VIC), 2 μL of cDNA, and 8.3 μL of H_2_O. The experiment was performed on a CFX Connect Real-Time System (Bio-RAD, USA) with the following conditions: 50 °C for 2 min, 95 °C for 5 min, followed by 40 cycles of 95 °C for 20 s and 60 °C for 1 min.

### 2.7. Clinical Sample Tests

To verify the availability and reliability of the colordetect RT-LAMP assay, 54 clinical samples were subjected to parallel detection using the newly established colorimetric RT-LAMP assay and a previously described reference RT-qPCR method [1]. The PAAPeV virus and ddH_2_O served as positive and negative controls, respectively. Following detection, the concordance between the results from the two methods was assessed.

### 2.8. Statistical Analysis

All experimental reactions were performed in triplicate. All histograms were generated using GraphPad Prism 9 (San Diego, CA, USA). Statistical significances of Ct values and relative fluorescence unit in primers screen assays and optimization of reaction conditions were evaluated by two-tailed unpaired *t*-tests using GraphPad Prism. Experimental data were expressed as the mean ± standard error of the mean (SEM), and statistical significance was defined as follows: * *p* < 0.05, ** *p* < 0.01, *** *p* < 0.001, and **** *p* < 0.0001.

## 3. Results

### 3.1. Phylogenetic and Sequence Identity Analysis of the PAAPeV 5′UTR Region

**Figure 1:**
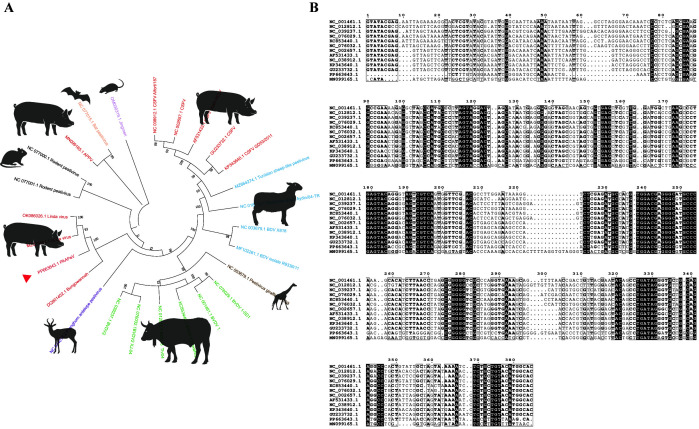
Phylogenetic and sequence identity analysis of the PAAPeV 5′UTR region. (**A**) Phylogenetic tree based on the 5′UTR nucleotide sequences of pestivirus members from NCBI was constructed using MEGA11 applying the Neighbor-Joining method for 1000 bootstrap replications. (**B**) 5′UTR nucleotide identity of PAAPeV compared with BVDV, CSFV, and APPV. Multiple sequence alignments of the 5′UTR nucleotide sequences of PAAPeV, BVDV, CSFV, and APPV were performed using ClustalX 2.1.

To determine the target sequence for designing colordetect RT-LAMP primers, phylogenetic analysis was performed using the 5′UTR nucleotide sequences of PAAPeV and 25 reference pestivirus sequences obtained from the NCBI GenBank database. The results demonstrated that PAAPeV clusters within a unique clade together with Linda virus strains (GenBank accession No.: OK086026.1; MZ027894.1) and the Bungowannah virus isolate (GenBank accession No.: DQ901402.1) (Figure 1A). Notably, the 5′UTR sequences of PAAPeV exhibit 76.3% nucleotide identity to Linda virus and 68.7% to Bungowannah virus. Conversely, all other representative pestiviruses, such as CSFV, BVDV, and APPV, form separate and distinct clades that do not cluster with PAAPeV (Figure 1A).

In addition, nucleotide identity analysis of the 5′UTR sequences was performed, which demonstrated that PAAPeV (GenBank accession No.: PP663643.1) exhibits 55.9–58.3% nucleotide identity to representative strains of BVDV types 1, 2, and 3 (GenBank accession No.: NC_076029.1; NC_001461.1; NC_076032.1; KC853440.1; NC_039237.1; NC_012812.1), 57.5–58.9% identity to CSFV strains (GenBank accession No.: NC_038912.1; NC_002657.1; GU233732.1; KP343640.1; AF531433.1), and 55.3% identity to APPV (Figure 1B). Consequently, LAMP primers were designed based on the 5′UTR of PAAPeV.

### 3.2. Colordetect RT-LAMP Assay Development and Primers Screen

Colordetect LAMP reactions were individually amplified using six pairs of RT-LAMP primers, with fluorescent indicators integrated for real-time monitoring and visual dyes incorporated to enable colorimetric interpretation. Resulting color changes were visually distinguishable: a red color indicated a negative result, while a yellow color denoted a positive result. Given that a shorter Ct value typically correlates with faster amplification kinetics of primer sets and enhanced detection sensitivity, and higher fluorescence intensity corresponds to a more obvious color change, all Ct values, fluorescence intensities, and colorimetric outcomes for each primer set were systematically recorded.

**Figure 2:**
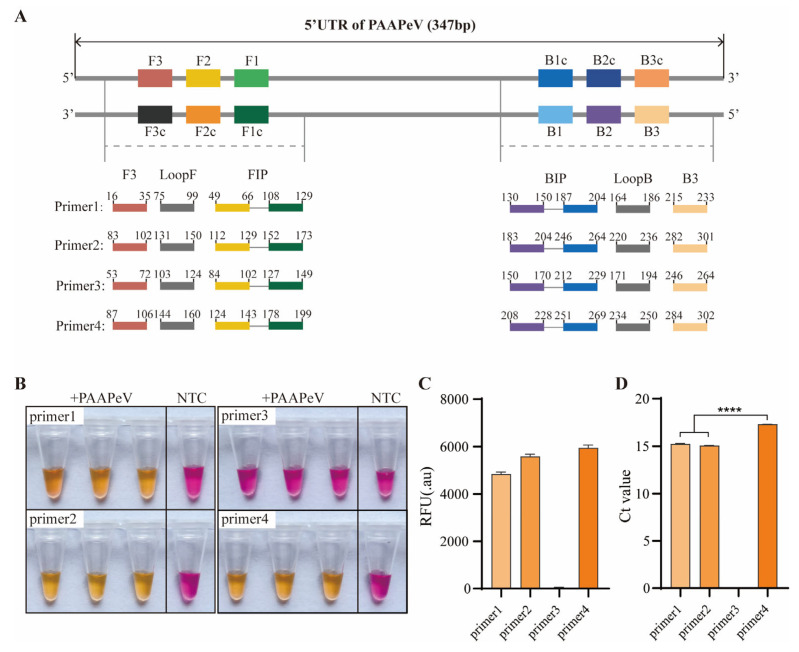
Colordetect RT-LAMP primer sets screen. (**A**) Graphical illustration of all primers designed for assay optimization. The end-point color change (**B**), relative fluorescence unit (RFU) (**C**) and cycle threshold (Ct) values (**D**) using different primer sets. NTC: no template control. Error bars represent SEM; *n* = 3, **** *p* < 0.0001.

To guarantee the specificity of the developed assay, four primer sets were prioritized, with their target sites showing substantial sequence divergence from the corresponding regions of BVDV, CSFV, and APPV. Among the evaluated primers (Figure 2A), three sets exhibited consistent amplification signals when PAAPeV RNA was used as the template. Primer sets 1, 2, and 4 produced distinct color changes in end-products; notably, the fluorescence intensities of primer set 1 and 2 were significantly lower than that of primer set 4 (Figure 2B,C). Furthermore, primer set 2 demonstrated the optimal performance, characterized by the earliest signal detection and the absence of background signals (Figure 2D). Consequently, primer set 2 was selected for subsequent experiments based on its superior amplification efficiency.

### 3.3. Optimization of Colordetect RT-LAMP Reaction Conditions

**Figure 3:**
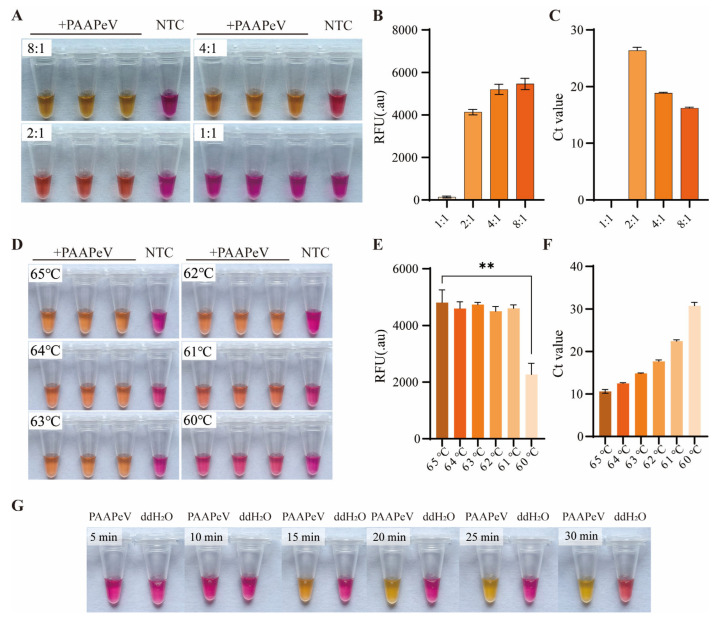
Optimal colordetect RT-LAMP reaction condition screening. The end-point color change, RFU and Ct values under different inner and outer primer ratios (1:1, 2:1, 4:1, 8:1) (**A**–**C**), reaction temperature (60 °C, 61 °C, 62 °C, 63 °C, 64 °C, 65 °C) (**D**–**F**), and reaction time (5 min, 10 min, 15 min, 20 min, 25 min,30 min) (**G**). NTC: no template control. Error bars represent SEM; *n* = 3, ** *p* < 0.01.

In order to determine the optimal inner/outer primer ratios, temperature and reaction time, the colordetect RT-LAMP was carried out using RNA of PAAPeV as a template. For primer ratio optimization, distinct color changes were observed in the end products at inner/outer primer ratios of 4:1 and 8:1. The 8:1 ratio yielded products with significantly higher fluorescence intensity and lower Ct values compared with the 4:1 ratio (Figure 3A–C). With respect to temperature optimization, the assay produced pronounced color changes in the end products across the range of 61–65 °C; fluorescence intensity measurements confirmed that 65 °C was the optimal incubation temperature for maximal assay performance (Figure 3D–F). For reaction time optimization, distinct color changes were detected after incubation for 15–25 min (Figure 3G). Taken together, these optimization experiments established that the refined colorimetric RT-LAMP assay displayed the most robust performance under the following optimized parameters: an inner/outer primer ratio of 8:1, an incubation temperature of 65 °C, and an incubation duration of 25 min.

### 3.4. Specificity of Colordetect RT-LAMP Assay

**Figure 4:**
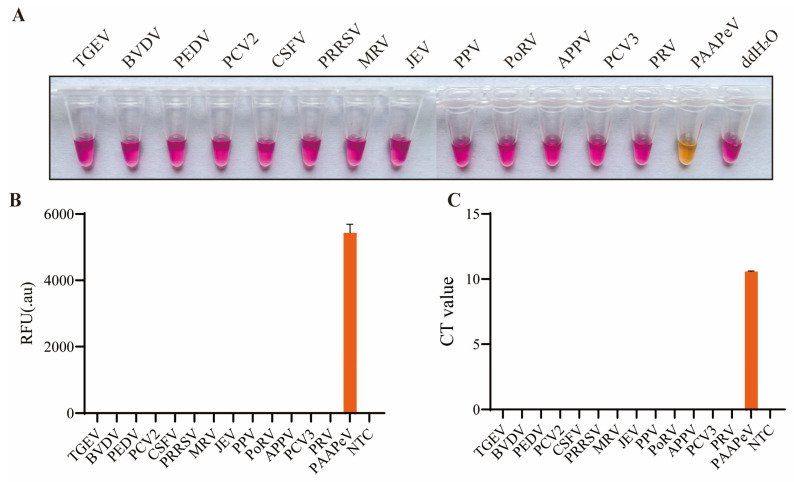
Specificity tests of colordetect RT-LAMP. The end-point color change (**A**), RFU (**B**) and Ct values (**C**) of colordetect RT-LAMP specificity testing. NTC: no template control.

To determine the specificity of the colordetect RT-LAMP assay, cross-reaction was performed with porcine and related viral pathogens, including TGEV, PEDV, PCV2, PRRSV (1 and 2), MRV, JEV, PPV, PoRV, PCV2, PCV3, PRV, BVDV, CSFV, and APPV. The PPAPeV and ddH_2_O served as positive and negative controls, respectively. Fluorescent signals and visual colorimetric changes were exclusively detected in the PAAPeV-positive control, whereas no positive reactions were observed in any of the other tested viruses or the ddH_2_O negative control (Figure 4). These results collectively demonstrate that the developed colordetect RT-LAMP assay exhibits excellent specificity for PAAPeV detection.

### 3.5. Sensitivity of Colordetect RT-LAMP Assay

**Figure 5:**
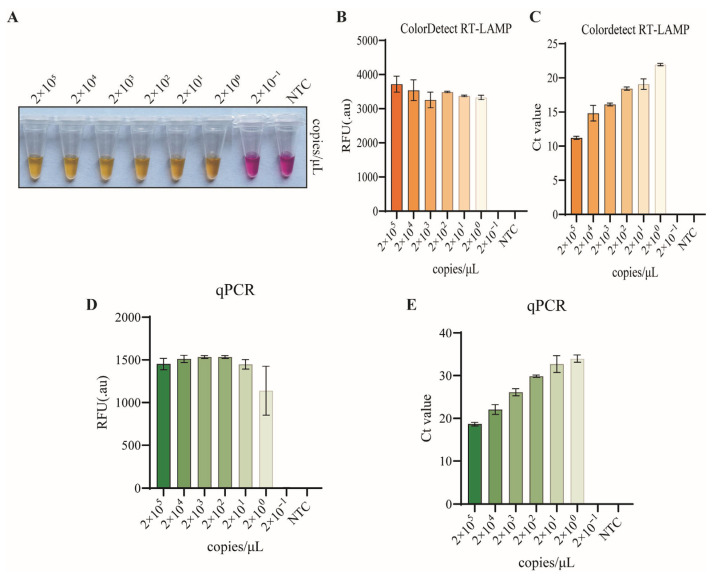
Sensitivity tests of colordetect RT-LAMP. A tenfold dilution series of pUC57-PAAPeV 5′UTR plasmid ranging from 2 × 10^5^ to 2 × 10^−1^ copies/µL were tested in duplicate using colordetect RT-LAMP (**A**–**C**) and qPCR (**D**,**E**). NTC: no template control. Error bars represent SEM; *n* = 3.

The sensitivity of the colordetect RT-LAMP assay was evaluated using 10-fold serially diluted pUC57-PAAPeV-5′UTR plasmids, with concentrations ranging from 2 × 10^5^ to 2 × 10^−1^ copies/μL. The results showed that distinct visual color changes were observed in the colordetect RT-LAMP reactions, and the lowest detection limit of the assay was determined to be 2 copies/μL (Figure 5A), which was consistent with the results of the fluorescence intensity and Ct value analyses (Figure 5B,C).

### 3.6. Clinical Concordance of Colordetect RT-LAMP and RT-qPCR

To determine the reliability of colordetect RT-LAMP assay for detection of viral RNA from clinical samples, a total of 54 clinical samples, including 24 clinical PAAPeV-positive samples and 30 clinical tissue samples (brains, lungs, spleen, and liver) derived from aborted fetuses and piglets exhibiting congenital tremors, were subjected to parallel detection using the established colordetect RT-LAMP assay and the gold-standard RT-qPCR method. The results indicated that all 24 clinical PAAPeV-positive samples, together with the positive control, tested positive by both colordetect RT-LAMP and RT-qPCR. In addition, 30 clinical tissue samples, as well as the negative control, yielded negative results with both assays. Collectively, these findings demonstrated 100% concordance between the two detection methods, with consistent identification of all PAAPeV-positive samples across both platforms (Table 2).

## 4. Discussion

Rapid and accurate diagnosis serves as the cornerstone for effective disease control strategies in both human and veterinary medicine [8]. Emerging porcine pestivirus-associated diseases continue to pose significant challenges to preventive and control measures within the global swine industry [22,23]. PAAPeV, a newly identified member of the genus *Pestivirus*, has been implicated in causing abortions in sows, as well as congenital tremors and neonatal mortality in piglets [1]. To date, RT-qPCR remains the only reported diagnostic method for PAAPeV detection. In this study, the newly developed colordetect RT-LAMP assay could achieve a detection limit of 2 copies/μL under 65 °C incubation for 25 min, using a set of six primers targeting the 5′UTR of the PAAPeV. Furthermore, this assay exhibits no cross-reactivity with other known pestiviruses or prevalent swine pathogens. Clinical validation demonstrated 100% concordance between the colordetect RT-LAMP assay and the gold-standard RT-qPCR method. Therefore, the developed colordetect RT-LAMP assay could serve as a rapid, sensitive, and specific diagnostic tool for resource-limited settings, and thus holds substantial promise for widespread deployment in on-site and regional veterinary laboratories.

RT-LAMP assays have been successfully developed for the detection of numerous RNA viruses, including avian influenza virus, classical swine fever virus, West Nile virus, and porcine reproductive and respiratory syndrome virus [14,17,19,24]. In this study, we established a colordetect RT-LAMP diagnostic assay for PAAPeV, which was simple to operate and provided immediate visual detection result with naked eyes, eliminating the need for specialized equipment. Like RT-PCR, the RT-LAMP technique amplifies the target viral RNA sequence under an invariable temperature between 63 °C and 65 °C [25]. In contrast to conventional methods (e.g., RT-PCR and RT-qPCR) that require specialized thermal cyclers, the colordetect RT-LAMP is executed in a single tube and requires only a simple water bath or heating block to provide a constant temperature of 65 °C for 25 min. Similarly, the BVDV RT-LAMP assay amplified a 228 bp target sequence of the 5′UTR of BVDV after incubation at 63 °C in 60 min [6] and the CSFV RT-LAMP reaction could be finished in 60 min under isothermal conditions at 65 °C by employing a set of four primers targeting the 5′ UTR of CSFV [7]. In contrast, RT-qPCR methods spend more than 1 h to complete the entire reaction [26,27].

The colordetect RT-LAMP assay established in this study exhibits excellent specificity and sensitivity. The 5′UTR is widely recognized as a critical target for viral detection and strain classification [3,28]. Notably, RT-LAMP assays targeting the 5′UTR have been successfully developed for the specific detection of CSFV and BVDV Oregon CV24, the minimum detection limit of which was 5 copies and 4.67 copies per reaction, respectively [6,7]. Similarly, the colordetect RT-LAMP assay, which also targets the 5′UTR of PAAPeV, achieved a detection limit of 2 copies per reaction at a constant temperature of 65 °C. Cross-reactivity testing revealed no non-specific reactions with nucleic acids from other known pestiviruses (CSFV, BVDV and APPV) or common swine pathogens (TGEV, PEDV, PCV2, PRRSV, MRV, JEV, PPV, PoRV, PCV3, PRV). Furthermore, in clinical sample validation, the detection results of our established assay showed complete concordance with the gold-standard RT-qPCR method, which further confirms the reliability and practical applicability of this colordetect RT-LAMP assay. These results are consistent with previous reports [6,7,29,30,31].

The high efficiency, operational simplicity, and low instrumentation dependence of colordetect RT-LAMP assay render it particularly advantageous for PAAPeV detection in resource-limited and remote swine-farming regions, addressing the practical limitations of current diagnostic methods. But aerosol contamination still poses a notable challenge during sample transfer processes in the RT-LAMP reaction workflow. To reduce the risk of contamination, the developed colordetect RT-LAMP assay, which incorporates Bst DNA polymerase, reverse transcriptase, and an optimized buffer solution, is designed for single closed-tube operation with the sole addition of specific primers and templates. Furthermore, all experimental procedures in the present study were rigorously executed in compliance with the standard operating guidelines for four distinct, physically separated working zones: the reagent preparation area, sample processing area, amplification area, and amplification product analysis area [32]. Even so, these shortcomings do not affect the robust performance of the colordetect RT-LAMP assay.

## 5. Conclusions

In conclusion, a colordetect RT-LAMP assay for the detection of PAAPeV has been established, which serves as a rapid, sensitive, and specific diagnostic tool for PAAPeV detection in resource-limited and remote swine-farming regions, thereby facilitating timely intervention to prevent the outbreak and transmission of its infections. In future research, we will continue to refine this assay by optimizing reaction conditions, simplifying operational steps, and integrating multiple technologies for the detection of a broader spectrum of pathogens.

## Figures and Tables

**Table 1:** The sequence of colordetect RT-LAMP primers.

Primers	Sequences (5′-3′)
1-F3	TGGGGATGCCAAAAACTGAA
1-B3	CGTCCCTAACACTGTGTCG
1-FIP	CAATGCAGCCTGCTCACCTACTAGGCCGAGGAAAGGTAGC
1-BIP	TGTAAGCGGTGAGTACACCGCCGTCCACGAGGCATAGCT
1-LoopF	CCACCTATCGAGTTCGTCCTACTAA
1-LoopB	GCTACTGGTAAGGATCACCCACT
2-F3	ACGAACTCGATAGGTGGACT
2-B3	CCGGCATCCTATCAGACTGT
2-FIP	TACCAGTAGCACCTCTGACGGCGGTGAGCAGGCTGCATTG
2-BIP	CACTAGCTATGCCTCGTGGACGTCACCCCCACTTTCAGGAC
2-LoopF	GCGGTGTACTCACCGCTTAC
2-LoopB	CAGTGTTAGGGACGGCG
3-F3	CGAGGAAAGGTAGCCAATCC
3-B3	TCACCCCCACTTTCAGGAC
3-FIP	CGGTGTACTCACCGCTTACACAACGAACTCGATAGGTGGACT
3-BIP	CAGCCGTCAGAGGTGCTACTGCCTAACACTGTGTCGGGC
3-LoopF	CAGCCTGCTCACCTACTAAACC
3-LoopB	GTAAGGATCACCCACTAGCTATGC
4-F3	ACTCGATAGGTGGACTGGTT
4-B3	GCCGGCATCCTATCAGACT
4-FIP	ACGAGGCATAGCTAGTGGGTGAGCATTGTGTAAGCGGTGAGT
4-BIP	GCGTGCCCGACACAGTGTTAGGGTATTCACCCCCACTTTC
4-LoopF	TCTGACGGCTGCGGTGT
4-LoopB	GCGGGGGCTGTCGTCCT

**Table 2:** Comparison between colordetect RT-LAMP assay and RT-qPCR.

Sample Source	ColorDetect RT-LAMP (Positive/Total)	RT-qPCR (Positive/Total)
Clinical PAAPeV-positive samples	24/24	24/24
Clinical unknown samples	0/30	0/30
Positive control	3/3	3/3
Negative control	0/3	0/3
Total	60	60
Diagnostic accuracy	100%	100%

## Data Availability

The original contributions presented in this study are included in the article. Further inquiries can be directed to the corresponding author.
